# Stimuli Responsive Poly(Vinyl Caprolactam) Gels for Biomedical Applications

**DOI:** 10.3390/gels2010006

**Published:** 2016-01-25

**Authors:** Kummara Madhusudana Rao, Kummari Subba Venkata Krishna Rao, Chang-Sik Ha

**Affiliations:** 1 Department of Polymer Science and Engineering, Pusan National University, Busan 609 735, Korea; msraochem@gmail.com (K.M.R.); 2 Department of Chemistry, Yogi Vemana University, Kadapa 516 003, Andhra Pradesh, India; drksvkrishna@yahoo.com (K.S.V.K.R.); 3 Department of Chemical Engineering and Material Science, Wayne State University, Detroit, MI48202, USA

**Keywords:** stimuli responsive, gels, poly(vinyl caprolactam), biomedical applications

## Abstract

Poly(vinyl caprolactam) (PNVCL) is one of the most important thermoresponsive polymers because it is similar to poly(N-isopropyl acrylamide). PNVCL precipitates from aqueous solutions in a physiological temperature range (32–34 °C). The use of PNVCL instead of PNIPAM is considered advantageous because of the assumed lower toxicity of PNVCL. PNVCL copolymer gels are sensitive to external stimuli, such as temperature and pH; which gives them a wide range of biomedical applications and consequently attracts considerable scientific interest. This review focuses on the recent studies on PNVCL-based stimuli responsive three dimensional hydrogels (macro, micro, and nano) for biomedical applications. This review also covers the future outlooks of PNVCL-based gels for biomedical applications, particularly in the drug delivery field.

## 1. Introduction

The development and applications of novel biomaterials play an important role in improving the treatment of diseases and the quality of health care. The focus of recent research on polymeric biomaterials is on producing new materials, including those with improved biocompatibility, mechanical properties and responsiveness. Polymeric biomaterials have been applied to medicine which includes controlled drug delivery systems, coatings of tablets, artificial organs, tissue engineering, polymer-coated stents, dental implants, and sutures [1,2]. This is a major step towards the development of polymeric therapeutic devices from newly synthesized polymeric materials with desired properties.

### 1.1. Stimuli Responsive Gels

The ability to swell and shrink in the presence and absence of aqueous media, respectively, is the most characteristic property of hydrogels [3]. Two important factors affect the swelling behavior of hydrogels; the hydrophilicity of the polymer chains and the crosslink density [3]. Hydrogels with stimuli responsive properties can be prepared by incorporating some stimuli-responsive co-monomers, either into the backbone of the network structure or as pendant groups. These hydrogels have the ability to swell, shrink, bend, or even degrade as a response to an external signal [3]. These stimuli-responsive hydrogels are also called “intelligent hydrogels”. They swell and shrink reversibly with small changes in the environmental conditions, such as pH, temperature, electric field, ionic strength, and type of salt [3].

Hydrogels have been used extensively in the development of smart delivery systems. In these systems, a polymeric matrix can protect a drug from hostile environments (such as low pH and enzymes), while controlling drug release when the gel structure is changing in response to environmental stimuli [4,5,6,7]. Bioactive molecules can be trapped easily by an equilibrium swelling method or by simply mixing before the formation of hydrogels. The drug can be released via a range of mechanisms triggered by various stimuli [3]. The recent interest in these systems has focused on hydrogels in the form of macro-, micro-, and nanogels because of their promising applications in drug delivery systems, cell encapsulation and enzyme immobilization [8,9]. Temperature-responsive hydrogels especially in micro or nano forms show a volume phase transition at a specific temperature that causes a sudden change in the solvent state. The incorporation of thermoresponsive polymers with other responsive moieties can exhibit dual responsive properties. The present review focuses mainly on the recent progress of the thermoresponsive behavior of PNVCL and their stimuli responsive gels for biomedical applications.

### 1.2. Importance of Thermoresponsive Poly(Vinyl Caprolactam)(PNVCL)

**Figure 1:**
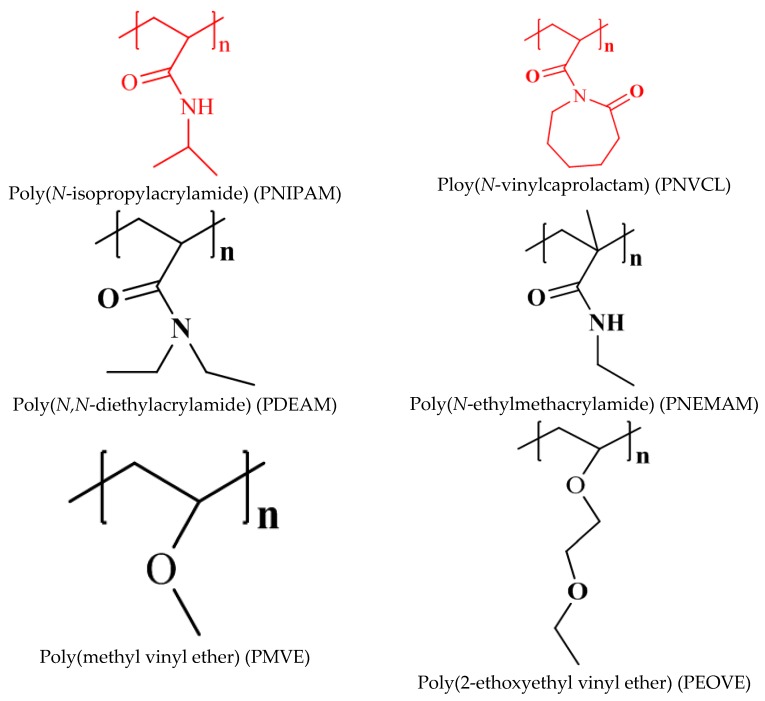
Chemical structures of the thermoresponsive polymers.

Most polymers are soluble in aqueous media when heated, but some water soluble polymers may precipitate from the solution upon heating [10]. This unique property is characteristic of polymers that dissolve when cooled and phase separate when heated above a certain temperature, known as a lower critical solution temperature (LCST) [10,11]. The LCST is mainly dependent on the hydrogen bonding between water molecules and the structure of functional monomer units of polymers. Phase or reversible conformational changes take place for thermosensitive polymers over small variations of temperature [11]. Various methods to determine sol-gel transition or phase change have been known, including cloud point measurement [12,13,14], differential scanning calorimetry (DSC) [15] and rheology [12]. The value of the transition temperature may vary slightly depending on the experimental method used, as different stages of the gelation process may be measured by each technique [14]. The most important thermoresponsive polymer is poly(*N*-isopropylacrylamide) (PNIPAM) [16,17,18,19], which undergoes a sharp coil-to-globule transition in water at 32 °C. Below this temperature, a hydrophilic state changes to a hydrophobic state. Water molecules associated with the side chain isopropyl moieties are released into the aqueous phase when temperature increases above the critical point. PNIPAM is easily accessible by radical polymerization with variable architectures such as block copolymers [20,21,22], gels [23,24,25] or grafted surfaces [26,27]. Therefore, PNIPAM-based materials are excellent candidates for applications in biosensing, controlled delivery and tissue engineering [28]. Figure 1 lists the important thermoresponsive polymers and their structures. Compared to PNIPAM, some other *N*-substituted poly(acrylamide)s exhibit similar behaviors in aqueous solutions. For example, poly(*N*,*N′*-diethylacrylamide) (PDEAAM), poly(*N*-cyclopropylacrylamide) and poly(*N*-ethylacrylamide) have been reported displaying LCSTs in the range from 30 °C to 80 °C [29]. Vinyl ether monomers can also be used to generate thermosensitive polymers. For example, poly(methyl vinyl ether) (PMVE) has a transition temperature at 35 °C, which renders it an interesting candidate for biomedical applications [30].

*N*-vinyl caprolactam (NVCL) has been known to possess noteworthy properties for biomedical applications, e.g., solubility in water and organic solvents, high absorption ability, and a transition temperature within the setting of these applications (33 °C) [31]. Even though the LCST of PNVCL solutions are close to PNIPAM, however, there are significant differences in the thermodynamic and molecular mechanisms underlying the phase transition. In contrast to PNIPAM, PNVCL possesses a classical Flory–Huggins thermoresponsive phase diagram. The phase transition behavior depends on the molecular weight of PVCL and the solution concentration [32,33]. This unique feature allows for controlling the temperature sensitivity of the polymer by varying its molecular weights. There are two stages in the phase transition of PNVCL in aqueous solution. The hydrogen bonding transformation is predominant at the first transition stage below the LCST and hydrophobic interaction is predominant at the second stage above the LCST [34]. A “sponge-like” structure may be formed for PVCL mesoglobules due to the absence of topological constrains as well as self-associated hydrogen bonds that could further continuously expel water molecules upon increasing temperature [32]. When the polymer chain length or polymer concentration is increased, the LCST of PNIPAM and PNVCL decreases [35,36]. In general, hydrophobic compounds decrease the LCST, whereas hydrophilic and charged compounds increase LCST of polymers due to the strong interactions between water and hydrophilic or charged groups [37]. LCST disappears when those polymers contain too many hydrophilic comonomers [36]. Salts are known to lower the LCST of PNIPAM and PNVCL [35,37,38,39]. Moreover LCST of PNVCL is known to decrease by addition of a small amount of alcohol [36]. Anionic surfactants usually prevent phase separation of PNVCL solutions when heating, while the hydrophobic interactions of anionic and cationic surfactants would lead them to bind to PNVCL. The behavior of PNVCL looks like a polyelectrolyte upon binding of the surfactant which means the polymer coil swells. As the surfactant concentration increases, the transition temperature increases [40]. Lau and Wu reported that the phase transition temperature of PNVCL depends on the molecular weight of the polymer [41]. Tager *et al.* showed for PNVCL with *M*w =5 × 10^5^ g/mol the change of the phase transition temperature from 32 °C to 34 °C depending on the concentration of the polymer in solution [42]. Meeussen *et al.* estimated the phase diagrams for polymers with different molecular weights. Moreover, cloud point measurements and theoretical calculations of PNVCL were carried out [31]. On the other hand, the lack of popularity among researchers for PNVCL compared to PNIPAM in previous years has likely been due to the affinity of polymerization of NVCL in a controlled manner because the polymerization kinetics is very difficult to measure. Unlike PNIPAM, the hydrolysis of PNVCL would not produce small amide compounds that are unwanted in biomedical applications [35]. Despite this, PNVCL have attracted attention for use in the biomedical field by considering the excellent properties such as biocompatibility [43]. This unique feature together with its overall low toxicity, high complexing ability and film forming properties enables its use in many industrial and medical applications, in particular in the biomedical field [34,35]. So far, various types of PNVCL based temperature responsive carriers such as micelles and vesicles have been reported and used for drug delivery applications [44,45,46,47]. However, the uses of hydrogels, especially in the form of micro/nanogels, are more advantageous in the biomedical field than other carriers since covalently crosslinked micro-/nanogels exhibit improved stability as compared to non-crosslinked carriers during *in vivo* delivery, leading to minimal premature release of bioactive agents [44,45,46,47]. However, chemical crosslinking also raises increasing concerns on the potential toxicity of those covalent crosslinking residuals. However, the purification step is a major concern to avoid such toxicity of hydrogels. In addition, the hydrogels can be used not only for drug delivery but also immobilize the enzymes or cells and are useful for biomedical applications.

### 1.3. Biocompatibility of PNVCL

Biocompatibility is the most important property in biomedical applications. PNVCL is not yet an FDA approved polymer. Henna *et al*. studied cytotoxicity of thermosensitive polymers like poly(*N*-isopropylacrylamide), poly(*N*-vinylcaprolactam) and amphiphilically modified poly(*N*-vinylcaprolactam). They have found that PNVCL is non-toxic in cellular contact during short exposure times, and enhanced cellular attachment is achieved with the PNVCL coated particles (compared to PNIPAM) [43]. Results show that PNVCL and PNVCL grafted with poly(ethylene oxide)(PEO) were well tolerated at all polymer concentrations (0.1–10 mg/mL) after 3 h of incubation at room temperature and at 37 °C. However, when the temperature increased to 37 °C (above LCST of PNVCL), cytotoxicity effects (8%) were also improved. In another study PNVCL-b-PEG was also found not to be toxic to human endothelial cells (E.A. hy EA.hy926) in the concentration range from 0 to 400 μg/mL [48]. The cytotoxicity of PNVCL, PNVCL-b-PVP on two cell lines (HeLa, human cervical carcinoma cells; and HEK293, human embryonic kidney cells) were negligible for all the tested samples for both cell lines compared with the negative control for polymer concentrations between 0.1 and 1 mg/mL [49].

## 2. Stimuli Responsive Poly(Vinyl Caprolactam) Gels

### 2.1. Macro Hydrogels

Stimuli responsive NVCL gels are formed using physical or chemical crosslinking methods. In the physical crosslinking method, the gelation behavior of a polymer solution can be affected by a range of factors, such as temperature, polymer concentration and type of solvent used. Chemical crosslinking involves covalent bonding through polymeric network chains. Recently, PNVCL-based gels have become important because of their biocompatibility and temperature responsive nature, highlighting their potential in the biomedical field [43]. As an important step towards a fundamental understanding of PNVCL gels and their potential applications, Andy *et al.,* synthesized a high molecular mass of PNVCL-based linear chains by free radical bulk polymerization in the presence of 2,2′-azobisisobutyronitrile (AIBN) as an initiator. The LCST of PNVCL is approximately 31.5 °C, which is close to the shrinking temperature of the PNVCL hydrogel [41]. PNVCL hydrogels were produced by free radical polymerization weakly crosslinked with *N, N′*-methylenebisacrylamide (MBA) as a crosslinker using an AIBN initiator [50]. The resulting gels have thermoresponsive behavior and the collapse is induced approximately at the same physiological temperature as for PNIPAM gels. The PNVCL gel transition depends on the surfactants and additives.

Boyko *et al.*, examined the gelation process on a radical chain crosslinking reaction based on NVCL by dynamic light scattering (DLS) [51]. In this study, free radical polymerization was performed using 3,3’-(ethane-1,1-diyl)bis(1-vinyl-2-pyrrolidone) (BISVP) as a crosslinker in the presence of AIBN as an initiator. The crosslinked gels followed the power law exponent, which is dependent on the concentration of the crosslinker. As the amount of crosslinker increases in the feed ratio of the gel, the network density increases with a higher exponent (μ). The power law behavior of PNVCL gels was found precisely at the sol–gel transition state and not in the gel state [51].

The strength of the PNVCL gels is also very important for applications. Morget *et al.* reported the tensile properties of PVCL gels, which were synthesized using ethylene glycol dimethacrylate and AIBN as the initiator [52]. The thermoresponsive gels showed that the stress-strain behavior was qualitatively different in the collapsed state above the temperature-induced system. At higher temperatures, the gels were stiffer, more ductile and showed greater time dependence.

The combination of thermoresponsive monomer, NVCL, with a pH responsive monomer yielded doubly responsive copolymers. Mamytbekov *et al.,* examined the swelling and mechanical properties of ionized networks of copolymers of NVCL and *N,N*-diallyl-*N*,*N*-dimethyl ammonium chloride (DMD) using BISVP as a crosslinker in the presence of gamma irradiation [53]. Upon heating, the system exhibited a continuous decrease in swelling with increasing volume phase transition temperature. Unlike other ionic hydrogels, the polyelectrolyte system showed continuous deswelling curves with increasing temperature. The degree of swelling in water and NaCl solutions was analyzed theoretically using the swelling equilibria of the polyelectrolyte network. The incorporation of vinyl pyrrolidine (VP) chains into these ionized networks showed a continuous phase transition from an expanded to collapsed state [54]. The transition temperature increased with an increasing amount of VP chains in the gels because the VP networks helped improve the hydrophilicity of the networks.

Interpenetrating polymer networks (IPNs) are a class of polymer blends, in which two polymeric networks are independently cross-linked. Lenka *et al.,* developed IPN gels of PNVCL and PNIPAM [55]. In this study, the PNVCL network was prepared using an ethylidene-bis-3(*N*-vinyl-2-pyrrolidone) (EBVP) crosslinker. Subsequently, NIPAM and MBA crosslinkers were polymerized in the swollen network structure of PNVCL using photo initiation exposure to UV light. In addition, they examined the volume phase transitions of IPN hydrogels using a range of techniques, such as nuclear magnetic resonance (NMR) spectroscopy, small angle neutron scattering (SANS), DSC, and dynamic mechanical measurements.

In the semi-interpenetrating polymer networks (semi-IPNs), one component of the assembly is crosslinked leaving the other in a linear form. Vyshivannaya *et al.,* developed a semi-IPN hydrogel using PAAm and PNVCL [56]. In their study, scattering experiments were used to examine the mechanism of the turbidity of thermoresponsive PNVCL and non-thermoresponsive poly(acrylamide) (PAAm) polymers and their mixture forming semi-IPNs. The clustered aggregates of PNVCL globules formed in the sols at high temperatures showed an improved contribution of the scattering due to clusters, whereas in the case of semi-IPNs, an increase in both the dynamic and static constituents of the intensity was observed.

The incorporation of cationic and anionic monomers further showed double (pH and thermo) the responsive behavior of the PNVCL hydrogels. Elcin Cakal *et al.*, synthesized a copolymeric hydrogel using a NVCL thermoresponsive monomer with 2-(diethylamino)ethyl methacrylate (DEAEMA) as a cationic monomer in the presence of free radical polymerization at 60 °C [57]. In this system, two important crosslinkers were used and equilibrium swelling was investigated with respect to both the pH and thermoresponsiveness. The pH sensitivity was strongly dependent on the DEAEMA content. Fickian swelling behavior was observed at pH 7.4 when the system contained more than 95 mol % of NVCL in the presence of both ethylene glycol dimethacrylate (EGDMA) and allyl methacrylate (AMA) crosslinkers. In another study, a cationic monomer, *N*-acryloyl-*N*’-ethyl piperazine (AcrNEP), was used for the synthesis of copolymeric hydrogels with NVCL in the presence of free radical polymerization at 75 °C with MBA as the crosslinker. The resulting gels were flexible and responsive to external stimuli with respect to both pH and temperature. They also examined the effects of the crosslinker on the properties of gel stimuli response, water transport mechanism, diffusion, and adsorption of anionic dyes [58].

Selva *et al.,* synthesized poly(*N*-vinylcaprolactam-*co*-itaconic acid) poly(NVCL-*co*-IA) gels in ethanol using the free radical crosslinking polymerization method at 60 °C for 24 h in the presence of AIBN and AMA as the initiator and crosslinking agent, respectively [59]. The incorporation of anionic monomer into the thermoresponsive PNVCL gels exhibited dual pH and temperature responsive behavior. They investigated the swelling behavior with respect to both pH and temperature. Further, swelling kinetics was also performed to know the diffusion mechanism of liquid from the diffusion exponent value (n). In this work, the n values for 5 and 10 mol % of itaconic acid (IA) present in the gels are 0.64 and 1.07, respectively. Based on these n values, the diffusion strongly depends on the IA content. The lower IA gels followed a completely non-Fickian pattern, whereas at higher content of IA, the mechanism changed from non-Fickian to case II. The relaxation controlled transport mechanism is highly dependent on the higher amount of ionizable IA groups for ionic gels. The incorporation of ionic monomers, such as acrylic acid (AAc) and methacrylic acid (MAAc), into physical gels of PNVCL also resulted in pH and temperature responsive behavior. Heba *et al.,* synthesized physically-crosslinked gels based on NVCL and AAc/MAAc via free radical polymerization [60]. The gels of the phase transition temperature was dependent on the solution pH because of the dissociate behavior of –COOH groups in the buffer solution. In addition, the incorporation of *N,N*-dimethylacrylamide (DMAm) into the system further increased the LCST. This increasing behavior of LCST was attributed to the increasing hydrophilicity of the system.

Zavgorodnya, *et al.* studied temperature-responsive properties of PNVCL multilayer hydrogels in the presence of Hofmeister anions [61]. The hydrogels were produced by glutaraldehyde-assisted crosslinking of hydrogen-bonded multilayers of PNVCL-*co*-(aminopropyl)methacrylamide) and poly(methacrylic acid). The authors found that swelling and temperature-induced shrinkage of PNVCL hydrogels were suppressed in the order SO_4_^2−^ > H_2_PO_4_^−^ > Cl^−^, following the Hofmeister series. In contrast, I^−^ increased hydrogel swelling but suppressed thermal response. Further, an optical response is initiated in the presence of anions when a layer of gold nanoparticles that has been stabilized with glutathione is introduced within the PNVCL hydrogel. When the temperature reversibly changed from 20 °C to 50 °C, the signal intensity of (PNVCL)_81_-Au hydrogels and the plasmon band position were remarkably dependent on ion concentration and type. These results are essential to understand the effect of Hofmeister anions on ultrathin non-ionic polymer networks. In addition, a distinct and fast optical monitoring of hydrogel temperature-triggered response depending on ion concentrations can be possible for the (PNVCL)_81_-Au hybrid hydrogels.

Sanna, *et al.* synthesized cellulose nanocrystals reinforced MBA crosslinked with PNVCL hydrogels using free radical polymerization method in the presence of trihexyltetradecylphosphonium persulfate (TETDPPS). The resulting nanocomposite hydrogels showed LCST of 33–34 °C. Rheological studies showed a significant increase of the mechanical properties even at very low CNC concentrations [62].

### 2.2. Micro or Nanogels

Sometimes, hydrogels can be confined to smaller dimensions, or exist in the form of macroscopic networks, such as microgels, which are crosslinked polymeric particles. When the microgels exist in the submicron range size, they are usually called nanogels [63,64,65]. Yinbing *et al.* examined the interaction of surfactant and PNVCL microgels [66]. In this study, the authors successfully prepared narrowly-distributed spherical PNVCL microgels by precipitation polymerization. The swelling and shrinkage of microgels were investigated by dynamic laser light scattering (LLS) with both anionic sodium dodecyl sulphate (SDS) and cationic *N*-dodecylpyridinium bromide (DPB) surfactants. The results show that the microgels gradually shrank to a collapsed state when the temperature was increased from 20 to 38 °C. The effects of the surfactant on the swelling and shrinking of the PNVCL microgels was attributed partially to the formation of micelles inside the microgel structure. The combination of hydrophilic monomer VP with NVCL produced a stable dispersion of larger particles by precipitation polymerization [67]. The microgels showed a strong decrease in the radius of gyration, *R_g_*, and hydrodynamic radius, *R_h_*, during the phase transition near 30 °C. The smooth surface of the macrogels indicates the decoration of hydrophilic polymer segments on the surface of the microgels. In another study, amphiphilic macro monomer polyethylene oxide (PEO) segments were used to prepare the thermally responsive microgels with NVCL using a macro monomer technique [65]. In this study, the polymerization yield was low because the potassium persulphate (KPS) initiator can hydrolyze the monomer NVCL. On the other hand, the microgels showed monomodal and narrow particle size distribution. Dispersion polymerization was used for the production of vinyl imadazole (Vim) functionalized poly(N-vinyl caprolactam-*co*-acetoacetoxy methacrylate) P(NVCL-*co*-AAEM) copolymer microgels [68]. A narrow size distribution with a hydrodynamic radius in the range 200–500 nm was obtained. The microgels exhibited both pH and thermo responsive behavior. The swelling property of the microgels depends on the VIm content in the microgel structure.

Crosslinking is also one of the important factors for the formation and their swelling characteristics of PNVCL microgels. Ainara *et al.* examined the formation of PNVCL microgels using different crosslinkers polyethyleneglycol diacrylate (PEGDA) and MBA and different concentrations [69]. The results showed that polymerization was complete within 10 min. In this case, both crosslinkers were consumed faster than the monomer. The crosslinking concentration did not affect the final collapsed diameters. On the other hand, the use of a small amount of crosslinker resulted in unexpected swelling behavior at temperatures below 20 °C. Another study showed similar results in that the MBA crosslinker and 3-*o*-methacryloyl-1,2:5,6-di-o-isopropylidene-α-d-glucofuranose (3MDG) monomers achieved complete conversion, whereas NVCL was not consumed completely [70].

Spherical microgels were produced from the precipitation copolymerization of NVCL and sodium acrylate (NaA) in water at 60 °C [71]. The copolymerization of a few mole-percent of ionic NaA into the P(VCL-*co*-NaA) microgel can raise its volume transition temperature slightly and increase the extent of its swelling. At the transition temperature (32 °C), the PVCL chains become hydrophobic and insoluble. The intra-microgel complexation between Ca^2+^ and these –COO^−^ groups lead to the shrinkage of microgels but inter microgel complexation induces aggregation. A comparison of the linear copolymer chains and spherical microgels showed that the aggregation of linear chains was much more profound than that of the spherical microgels, presumably because of the competition between the inter- and intra-chain complexation. The same research group also examined Ca^2+^-induced complexation between the thermally responsive P(NVCL-*co*-NaA) microgels with gelatin linear chains in water. These results suggest that the microgel can form complexes with gelatin in the presence of Ca^+2^ and complexation is induced by both hydrophobic and electrostatic attractions. Gelatin absorbed effectively on the surface of the gel due to the above said mechanism [72].

## 3. Biomedical Applications

Table 1 summarizes recent significant studies on the stimuli responsive gels of PNVCL for biomedical applications.

### 3.1. Drug Delivery and Antibacterial Applications

Iskav *et al.* developed thermoresponsive PNVCL hydrogels with sodium itacanote. The gels were used to encapsulate farmazine (Far) as a model release agent [73]. In this study, the phase transition temperature was shifted 2.5 °C to a lower temperature for the drug immobilized gels compared to the pristine gels. The authors attributed this shift to hydrogen bonding of the lactam oxygen atom of the polymer to the amino group of Far. The gels exhibited the controlled release of the drug via diffusion. The half time of drug release was 32 min at 22 °C and 210 min at 37 °C.

**Figure 2:**
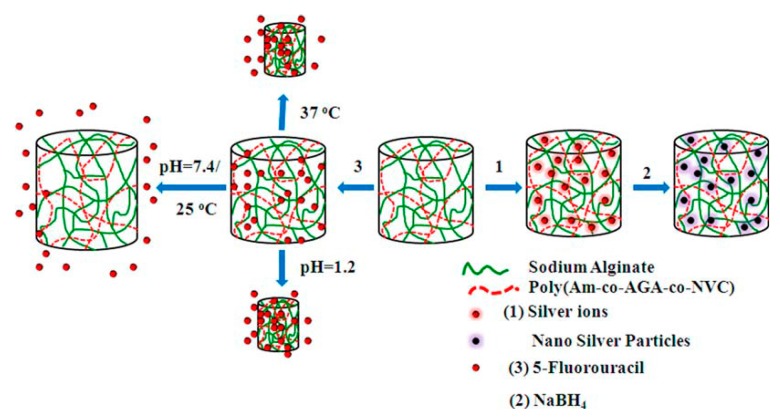
Synthesis and dual responsive-triggered release of the drug from semi-Interpenetrating Polymeric Networks (semi-IPNs) of SAlg-P(AAm-*co*-AGA-*co*-NVCL) multi component gels and the formation of Ag NPs in the hydrogel networks. (Reproduced from Rama Subba Reddy *et al.,* *Macromol. Res.* **2014**, *8*, 832–842. (Ref. 74) copyright 2014 with permission from Springer).

Reddy *et al.,* synthesized a dual responsive semi-IPN hydrogels using sodium alginate (SAlg) and poly(acrylamide-*co*-acrylamidoglycolic acid-*co*-*N*-vinyl caprolactam) P(AAm-*co*-AGA-*co*-NVCL) multi components via free radical redox polymerization [74]. In this study, 5-FU as a model anticancer agent, was used for encapsulation into these semi-IPNs via an equilibrium swelling method. In addition, the authors synthesized silver nanoparticles (Ag NPs) with a size and shape-controlled spherical shape and an average size of approximately 20 nm in the gel networks by simple equilibration of the Ag salts and reduced in the presence of NaBH_4_ (Figure 2). The authors proved that the gels could control the release of the drug and Ag NPs through stimuli, such as pH and temperature. As shown in Figure 2, the temperature-responsive drug release mainly caused the presence of PNVCL copolymeric chains in the gel networks.

**Figure 3:**
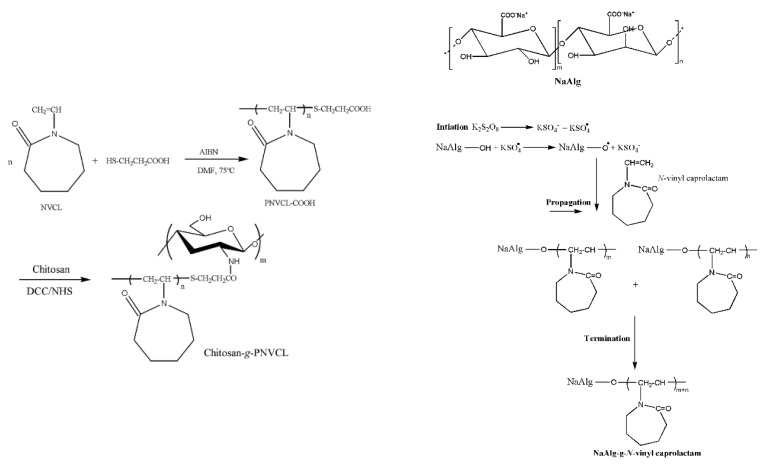
Synthesis of the graft copolymers of CS and SAlg with NVCL [75,77]. (Reproduced from Madhusudana-Rao *et al.*, *J. Appl. Pharm. Sci.* **2013**, *3*, 061–069(Ref. 75) copyright 2013 with permission from Journal and Prabaharan *et al.*, *Macromol. Biosci*. **2008**, *8*, 843–851 (Ref. 77) copyright 2008 with permission from John Wiley & Sons, Inc.).

The incorporation of a NVCL thermoresponsive monomer on the backbone of biopolymers, such as chitosan (CS) and SAlg, by grafting could improve the properties without sacrificing the biodegradable nature. As shown in Figure 3, grafting was achieved via the free radical polymerization of NVCL or the coupling reaction of PNVCL onto SAlg or CS. Rao *et al.* prepared microgels using a PNVCL-g-SAlg graft polymer via an ionotropic gelation method. The effects of pH and temperature on the swelling behavior of microgels studied ascertained that they were sensitive to pH and thermoresponsive properties [75]. The mean size of the microgels was approximately 100 μm with a smooth and spherical shape. 5-fluorouracil (5-FU), as the model anticancer drug, was loaded and the encapsulation efficiency was found to be 84%. The authors systematically examined the release of 5-FU as a function of temperature, pH, amount of crosslinker, and % of drug concentration. The gels exhibited controlled release behavior over a period of more than 12 h. In another study, the same graft copolymer was used to produce the gel beads and utilized the encapsulation of the 5-FU model drug using GA as a crosslinker. The mean size of the gel beads was also close to 100 μm. The authors also examined the thermoresponsive behavior and release studies using a range of parameters [76]. Prabaharan, *et al.*, synthesized CS-g-PNVCL by grafting carboxyl-terminated PNVCL chains onto the CS backbone via a DCC coupling reaction and used it as a drug delivery carrier [77]. The MTT (3-(4,5-dimethylthiazol-2-yl)-2,5-diphenyltetrazolium bromide) assay showed no obvious cytotoxicity of CS-g-PNVCL against a human endothelial cell line over a concentration range of 0–400 μg/mL. The beads were crosslinked with TPP and encapsulated Ket as the model drug. Drug release was influenced by both pH and temperature. The authors concluded that the release was very slow at pH 7.4 and 37 °C because the gel beads had a compact structure with a reduced pore size and strong interaction between the drug molecules and polymer chains.

A graft copolymer of poly(N-vinyl caprolactam-g-PEO macromonomer (PNVCL-g-C_11_EO_12_) based gels was formed, physically crosslinked with salicylic acid (SA) via H-bonding interactions between PNVCL and SA [78]. In this study, the gel’s stability was dependent mainly on the phase transition temperature. At 23 °C, which is below the LCST, the gel stability was poor, whereas more stable hydrogels were formed above the LCST (37 °C). On the other hand, the graft copolymer provided more H-bonding interactions with SA, leading to sustained drug release compared to PNVCL.

The combination of metal nanoparticles with smart polymeric architectures appears to be a promising way of designing new materials. In this context, microgels are promising templates for the synthesis of Ag NPs in the networks. Nadine *et al.* reported that PNVCL based microgels were effective templates for loading of different concentrations (1–12 wt. %). The porous structure of the poly(*N*-vinyl caprolactam-*co*-glycedyl methacrylate) P(NVCL-*co*-GMA) microgels allowed the release of the AgNPs [79]. The Ag NP-embedded microgels exhibited good antibacterial activity against Gram-positive *S. aureus*, and Gram-negative *P. aeruginosa*.

The microgels were developed by the incorporation of undecenoic acid (UA) functionalized PNVCL which showed a double responsive phase transition temperature [80]. In this study, the LCST of the microgels was close to body temperature. Doxorubicin (Dox) as a model anticancer drug was encapsulated via a thermal gelation process. The release of Dox was dependent on the pH and temperature. Cytotoxicity of PNVCL, and their microgels (5% and 10% UA) were performed on 3T3 murine fibroblast cells using CCK-8 assay at concentrations from 0.15 to 10 mg/mL. The results confirmed no obvious toxicity to the cells. These results can be applied to the temperature and pH controlled delivery of anticancer drugs. Based on the intracellular environment of cancer cells, Yang *et al.,* reported intracellular degradable multi responsive microgels composed from NVCL and methacrylic acid (MAAc) from the disulphide methacrylate crosslinker by precipitation polymerization [81]. The microgels were spherical and degradable in response to GSH due to the availability of disulphide bonds in the networks of the microgels. The microgels could effectively encapsulate the Dox drug and exhibited stimuli-triggered drug release in acidic or reducing environments. Further, to confirm the biocompatibility of microgels, the authors tested cytotoxicity of microgels on HK-2 cells using MTT assay. After 48 h incubation, the microgels showed no cytotoxicity in the concentration range 0.1–50 μg/mL. Ninety percent of cells were viable at higher concentrations (100 μg/mL), also proving that the microgels are highly biocompatible. The cytotoxicity of DOX loaded microgels were evaluated against Hela cancer cells. The authors found IC50 (at a concentration 2.1 μg/mL DOX in the microgels) is slightly higher than free DOX (1.4 μg/mL). Further, the DOX loaded microgel studies of intracellular drug release on Hela cancer cells confirmed the high cell uptake and high anticancer activity.

**Figure 4:**
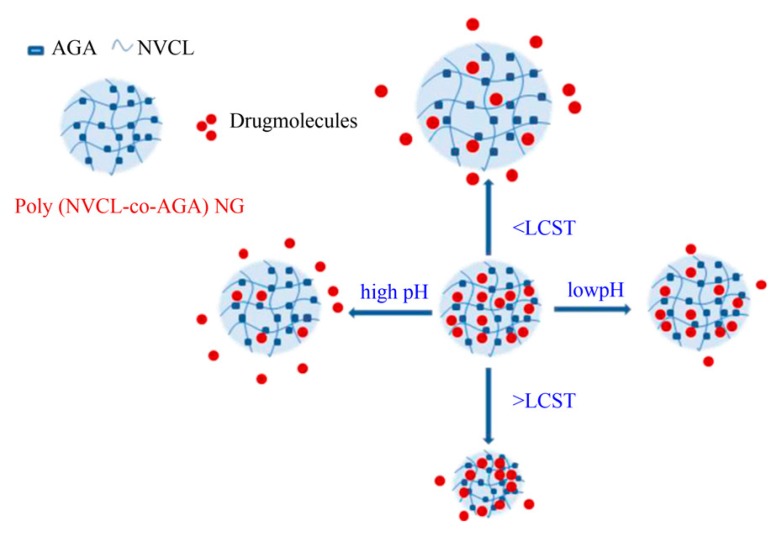
pH and temperature responsive nature of NGs [85]. (Reproduced from Madhusudana Rao *et al., Colloids Surf. B* **2013**, *102*, 891–897 (Ref. 87) copyright 2013 with permission from Elsevier).

**Figure 5:**
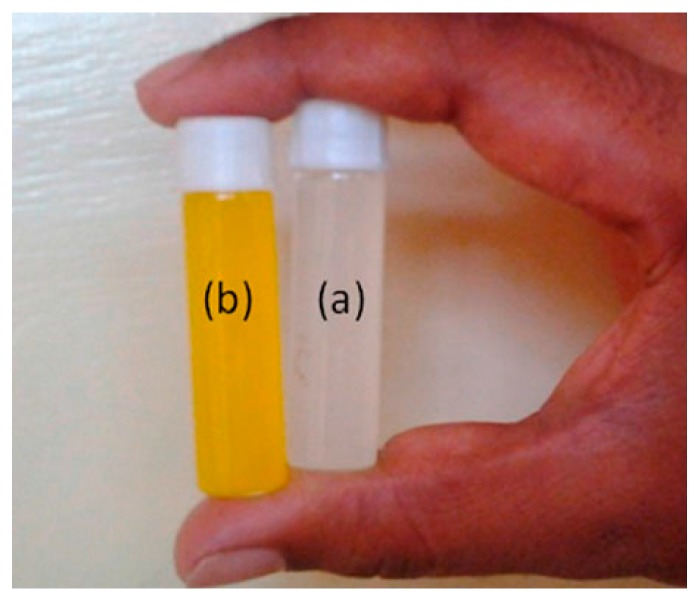
(**a**) Aqueous dispersions of NGs and (**b**) curcumin-loaded NGs. (Reproduced from Sudhakar *et al.*, *Des. Monomers Polym.* **2015**, *18*, 705–713. (Ref. 86) copyright 2015 with permission from Taylor and Francis).

Nanogel platforms are better for the loading and release of bioactive agents than other carriers [82,83] because of the stability, responsiveness and biocompatibility of nanogels. The particle size of the nanogels plays a crucial role in the *in vivo* fate of colloidal drug delivery systems [84]. Therefore, easy and effective control over the particle size is of great importance. Rao *et al.* developed copolymeric nanogels from NVCL and acrylamidoglycolic acid (AGA) crosslinked with MBA by emulsion polymerization [85]. In this method, the particle size of the nanogels produced was approximately 50–100 nm with a spherical shape. In addition, 5-FU model anticancer drug encapsulated and the controlled release of 5-FU was examined at different pH and temperatures. From *in vitro* release, the nanogels showed pH and thermoresponsive behavior (Figure 4). Sudhakar *et al.* used the same emulsion polymerization for the incorporation of hydrophilic 2-hydroxyethyl methacrylate (HEMA) chains into PNVCL nanogels and produced a spherical shape, 150 nm in size [86]. In this study, a hydrophobic curcumin (CUR) model drug was encapsulated successfully during the polymerization process. The nanogels showed high aqueous stability (Figure 5) and were more bioavailable. The *in vitro* release studies also proved that the resulting nanogels were thermoresponsive and useful for targeted drug delivery applications. In another report, Mirian *et al.,* incorporated polyethyleneglycol methacrylate (PEGMA) chains into PNVCL nanogels using surfactant-free emulsion polymerization [87]. In addition, vinyl pyrrolidine (VP), 2-methacryloyloxybenzoic acid (2MBA) monomers were also incorporated to adjust the phase transition temperature. The nanogels sizes are between 120 and 300 nm. The nanogels containing 15.5% 2MBA showed a transition temperature close to 38 °C. In this study, 5-FU was also used as a model bioactive agent. The release of 5-FU was slower at pH 7.4 and 37 °C than under the tumor cellular condition (pH 6.0 and 40 °C). Drug release kinetics studies also proved that the drug release from the nanogels followed Fickian diffusion.

**Table 1:** Summary of the recent significant studies on the stimuli responsive gels of poly(*N*-vinyl caprolactam) PNVCL for biomedical applications.

Gel Composition	Responsiveness		Release Carriers	Application	Reference
P(NVCL-*co*-SIA)	thermo	Hydrogel	Far	Controlled release	[73]
P(AAm-*co*-NVCL-*co*-AGA)/SAlg	pH/thermo	Composite hydrogel	5-FU/Ag nano	Controlled release/Antibacterial	[74]
SAlg-g-PNVCL	pH/thermo	Microgel	5-FU	Controlled release	[75]
SAlg-g-PNVCL	pH/thermo	Gel beads	5-FU	Controlled release	[76]
CS-g-PNVCL	pH/thermo	Gel beads	Ket	Controlled release	[77]
PNVCL-g-PEO	thermo	Gel particles	Nad/Prop/Ket/SA	Controlled release	[78]
PNVCL-*co*-GMA	thermo	Hybrid microgel	Ag	antibacterial	[79]
P(NVCL-*co*-UA)	pH/thermo	Microgel	Dox	Controlled release	[80]
P(NVCL-PMAA)	pH/thermo/reduction	Microgel	Dox	Tumor targeted drug delivery	[81]
P(NVCL-*co*-AGA)	pH/thermo	Nanogel	5-FU	Controlled release	[85]
PVOH-b-PNVCL	pH/thermo/reduction	Nanogel	NR	Tumor targeted drug delivery	[86]
P(NVCL-*co*-HEMA)	thermo	Nanogel	CUR	Targeted drug delivery	[87]
P(NVCL-*co*-PEGMA)/VP/2MBA	pH/thermo	Nanogel	5-FU	Controlled release	[88]
PNVCL/Dex-MA	pH/thermo/Enzyme	Nanogel	dextranase	Enzymatic degradation	[89]
Fib-g-PNVCL	Thermo	Nanogel	5-FU/Meg	Tumor targeted drug delivery	[90]
HPCL-click-PNVCL	Thermo	Films	---	controlled drug delivery	[91]
PVCL-NH_2_/PMAA	Thermo/pH	Gel cubes, capsules	----	controlled drug delivery	[92]
PNVCL	thermo	Hydrogel	Bacteria and fungi	Medicine	[93]
PNVCL-CaAlg	pH/thermo	Hydrogel	Enzymes and cells	Medicine	[94]
PNVCL-CaAlg	pH/thermo	Hydrogel	Protease	Biotechnology/medicine	[95]

Liu *et al.* reported the synthesis and loading/release properties of the reversibly-crosslinked thermo and redox responsive nanogels based on poly(vinyl alcohol)-g-*N*-vinyl caprolactam (PVOH-b-PNVCL) copolymers [88]. The heating and cooling cycles used for the formation of nanogels with 3,3′-dithiodipropionic acid (DPA) as crosslinker. In the presence of dithiothreitol (DTT) (10 mM), the gels exhibited degradation, and reversible crosslinking occurred upon exposure to H_2_O_2_. The authors used Nile red as the model compound to evaluate the release and cellular uptake. Cytotoxicity of PVOH-b-PNVCL copolymeric nanogels was tested against mouse fibroblast-like L929 cell line using *in vitro* culture via MTS (3-(4,5-dimethylthiazol-2-yl)-5-(3-carboxymethoxyphenyl)-2-(4-sulfophenyl)-2H-tetrazolium) assay. The nanogels exhibited high cell viability above 85%, even after 48 h incubation with 2 mg/mL concentrated nanogels. The results suggested the nanogels have low cytotoxicity. Further, their preliminary studies on cellular uptake of the nanogels within human melanoma (MEL-5) cells confirmed the cellar internalization of nanogels. Two important families of thermo responsive enzymatically degradable nanogels were developed by the batch emulsion polymerization of NVCL with dextran methacrylate (Dx-MA) [89]. The crosslinking nanogels were swollen below the volume phase transition temperature and collapsed above it. On the other hand, the low degree of crosslinked nanogels followed anomalous thermal behavior. The dextranase enzymatic degradation of the nanogels resulted in the release of sugars. Both families can be suitable for drug delivery in tissues or organs.

Like graft copolymeric microgels, Sanoj *et al.*, developed multi-drug loaded thermo responsive fibrinogen-g-*N*-vinyl caprolactam (Fib-g-PNVCL) nanogels and used for breast cancer drug delivery [90]. In this work, 5-FU and Meg multi drugs loaded into these nanogels. The LCST of the nanogels tuned by adjusting the composition of PNVCL/fib was approximately 35 °C. The sizes of the nanogels produced were 150–170 nm.The multi drugs are responsible for enhanced anticancer activity towards the MCF-7 cancer cells. The *in vivo* experiments also examined on Swiss albino mice showed the sustained release of Meg and 5-FU within three days.

Cai *et al*., synthesized hyperbranched poly(3-caprolactone) with peripheral terminal alkyne groups (HPCL) via thiol–yne click reaction among the AB_2_-type α-thiol-ω-alkyne–poly(3-caprolactone) linear precursors [91]. Azide-terminated poly(*N*-vinylcaprolactam) (PNVCL–N_3_), prepared *a priori* via xanthate mediated reversible addition–fragmentation chain transfer (RAFT) polymerization of NVCL, was then linked to HPCL chains through Cu(I)-catalyzed alkyne-azide click reaction. Further, the authors fabricated membranes using these polymers with well-defined and uniform pores. The swelling of HPCL-click-PNVCL membranes resulted in the temperature-responsive property. Cell viability studies using MTT assay on 3T3 fibroblast cell line incubated with copolymer membranes for 24 h resulted in more than 90% of cell viability. The cytotoxicity assays thus indicate that the introduction of PNVCL chains on the HPCL membrane and pore surfaces has negligible cytotoxicity effects and the non-cytotoxic nature of HPCL membranes. These stimuli responsive membranes with controllable morphology improved mechanical properties and negligible cytotoxicity, which are useful as biomaterials for controlled drug delivery.

Liang *et al*. reported a novel type of single-component PNVCL multilayer hydrogel films and capsules with a distinct and highly reversible thermoresponsive behavior derived from hydrogen-bonded multilayers of PMAA and PNVCL-NH_2_ by chemical crosslinking of PNVCL-NH_2_ copolymer layers and subsequent release of PMAA at basic pH values [92]. Cubical (PNVCL)_7_ hydrogel capsules retained their cubical shape when temperature was changed from 25 to 50 °C while showing a size decrease of 21% ± 1%. Spherical (PNVCL)_7_ hydrogel capsules demonstrated the similar shrinkage of 23% ± 1%. The temperature-triggered size changes of both types of capsules were completely reversible. Non crosslinked two component films did not show temperature-responsive behavior because of the presence of PMAA. This work opened up new prospects for developing biocompatible hydrogel-based nanothin coatings and shaped containers for temperature-regulated drug delivery, cellular uptake, sensing, and transport behavior in microfluidic devices.

### 3.2. Enzyme/Cell Immobilization

The PNVCL gels not only encapsulated the drugs, but also immobilized the cells and enzymes. Marina *et al.* developed bacteria and fungi microbial cells entrapped in thermoresponsive PNVCL gel beads. The results showed that the enzymatic activities of the microorganisms decreased after the entrapment of cells in the gel beads. This study allowed the preparation of gels of different cells as biocatalysts in a single step procedure for the transformation of both hydrophilic and lipophilic substrates [93]. A one step method was used to fabricate macroscopic poly(*N*-vinyl caprolactam-*co*-calcium alginate (PNVCL-CaAlg hydrogels) for the entrapment of human cells and enzymes, and their characteristics as immobilized enzymes including their thermal stability and storage stabilities were investigated. Three important proteases, *i.e.*, trypsin, α-chymotrypsin, carboxypeptidase B, and thrombin immobilized in hydrogels, could be used at 65–80 °C, whereas the native enzymes were completely inactivated at 50–55 °C. The hydrogel beads with entrapped α-chymotrypsin were used in the enantioselective hydrolysis of Schiff’s base of d,l-phenylalanine ethyl ester (SBPH) in an acetonitrile/water medium, thrombin immobilized in PVCL-based hydrogel films used for wound treatment. Hybridoma cell lines producing MAb to interleukin-2 were also cultivated successfully in the hydrogel beads [94]. Another study also used PVCL-CaAlg gels for the entrapment of trypsin and CPB. This method is advantageous for the immobilization of enzymes and their application in biotechnology and medicine [95].

## 4. Conclusions and Future Outlooks

The number of studies on PNVCL is currently low, at least compared to those on PNIPAM. In addition, PNVCL exhibits good biocompatibility and is attractive to the biomedical field. On the other hand, PNVCL is not yet FDA approved but there are an increasing number of reports highlighting its applicability in drug delivery and tissue-engineering fields. The limitation in the popularity of PNVCL polymerization was not controllable. Recently, however, controlled living polymerizations resolved this problem through the use of reversible addition–fragmentation chain transfer (RAFT) and macromolecular design via the interchange of xanthates (MADIX) polymerization (RAFT/MADIX) and cobalt-mediated polymerization methods [91,96,97,98]. This review summarized the synthesis and stimuli responsive behavior of PNVCL gels and their recent progress in biomedical fields, such as drug delivery, encapsulation of cells and the immobilization of enzymes. The incorporation of ionic moieties, such as cationic and anionic functional monomers, into thermoresponsive PNVCL gels made them pH and temperature responsive, making them suitable for drug delivery applications. In addition, pH, thermoresponsive and intracellular degradable micro/nanogels were also studied for cancer drug delivery. In future, the research based on PNVCL gels is expected to grow because, already, a few studies have confirmed the biocompatibility of PNVCL alone or in combination with other polymers. At present, a number of studies on micro/nano gels have been successfully applied in biomedical fields, especially in drug delivery. However, PNVCL-based hydrogels in tissue engineering has not yet been studied. In future, there is a need to develop methods and modifications with other polymers in order to improve the cytocompatability. Although stimuli responsive nanogels of PNVCL have already been reported, it will be necessary to develop new synthetic strategies to modify them with other functional groups, targeting ligands in the biomedical field, particularly cancer targeted drug delivery.

## Abbreviations

**Monomers**	**Crosslinkrs**
NVCL: *N*-vinyl caprolactam	BISVP: 3,3’-(ethane-1,1-diyl)bis(1-vinyl-2-pyrrolidone)
NIPAM: *N*-isopropyl acrylamide	DMD: *N,N*-diallyl-*N,N*-dimethyl ammonium chloride
DEAM: *N,N*-diethyl acrylamide	MBA: *N, N’*-Methylenebisacrylamide
MVE: methyl vinyl ether	EBVP: Ethylidene-bis-3(*N*-vinyl-2-pyrrolidone)
NEMAM: *N*-ethylmethacrylamide	EGDMA: Ethylene glycol dimethacrylate
EOVE: 2-ethoxyethyl vinyl ether	GA: Glutaraldehyde
NVIBAM: *N*-vinylisobutyramide	TPP: Tripolyphosphate
VP: vinyl pyrrolidine	**Polymers**
AAm: acrylamide	PAA: Poly(acrylamide)
DEAEMA: 2-(diethylamino)ethyl methacrylate	PEO: polyethylene oxide
AcrNEP: *N*-acryloyl-*N*’-ethyl piperazine	SAlg: Sodium Alginate
AMA: Allyl methacrylate	CS: Chitosan
IA: Itaconic acid	PVOH: Polyvinyl alcohol
AAc:Acrylic acid	Fib: Fibrinogen
MAAc: Methacrylic acid	PNVCL: Poly(*N*-vinyl caprolactam)
DMAm: *N,N*-dimethylacrylamide	PNIPAM: poly(*N*-isopropylacrylamide)
Vim: Vinyl imadazole	**Intiators**
AAEM: Acetoacetoxy metthacrylate	AIBN: Azobis(isobutyronitrile)
3MDG: 3-*o*-methacryloyl-1,2:5,6-di-*o*-isopropylidene-α-d-glucofuranose	ABIN: 2,2’-azobis(2-methylpropoionate)
AGA: Acrylamidoglycolic acid	KPS: Potassium persulphate
NaA: Sodium acrylate	APS: Ammonium persulphate
C_11_EO_12_:PEO macromonomer	**Surfactants**
UA: Undecenoic acid	SDS: Sodium dodecyl sulphate
2MBA: 2-methacryloyloxybenzoic acid	DPB: *N*-dodecylpyridinium bromide
Dx-MA: Dextran mehacrylate	
**Drugs**	**Others**
5-FU: 5-Fluorouracil	NaBH_4_: Sodium borohydride
Far: Farmazine	GSH: Glutathione
Dox: Doxorubicin	LCST: Lowest critical Solution temperature
Ket: Ketoprofen	LLS: Laser light scattering studies
CUR: Curcumin	DLS: Dynamic light scattering studies
Meg:Megestrol acetate	IPN: Interpenetrating Polymer Network
Nad: Nadolol	MTT: 3-(4,5-dimethylthiazol-2-yl)-2,5-diphenyltetrazolium bromide
Prop: Propranolol	RAFT: Reversible addition–fragmentation chain transfer
SA: Salicylic acid	
